# The role of long non-coding RNA LINC01296 in oral squamous cell carcinoma: a study based on bioinformatics analysis and *in vitro* validation

**DOI:** 10.7150/jca.60417

**Published:** 2022-01-01

**Authors:** Kaicheng Yang, Lei Li, Yanping Chen, Shasha Man, Lei Yang, Yinglai Yang, Naiheng Hei, Jianguang Zhao

**Affiliations:** 1 Department of Stomatology, the Fourth Hospital of Hebei Medical University, No.12 Jiankang Road, Shijiazhuang, Hebei province 050000, China.; 2 Department of Stomatology, the Third Hospital of Hebei Medical University, No.139 Ziqiang Road, Shijiazhuang, Hebei province 050000, China.; 3 Department of Stomatology, Shijiazhuang Second Hospital, No.53 Huaxi Road, Shijiazhuang, Hebei province 050000, China.; 4 Department of Stomatology, Wei County People's Hospital, No.361 Kaifangxi Road, Xingtai, Hebei province 054700, China.

**Keywords:** cell growth, LINC01296, long non-coding RNA, migration and invasion, oral squamous cell carcinoma

## Abstract

**Background:** Oral squamous cell carcinoma (OSCC) is a common malignancy in the oral cavity that represents a significant global health problem. Multivariate analysis has shown that long non-coding RNA LINC01296 plays an important role in cancer biology. However, the functions of LINC01296 in OSCC are still unknown.

**Methods:** The RNA profiles and clinicopathological information of OSCC patients and healthy subjects were downloaded. The expression level and prognostic value of LINC01296 were assessed. The functions and pathways of LINC01296were explored using the Gene Set Enrichment Analysis (GSEA) and functional analysis. The expression of LINC01296 in OSCC tissues and cell lines was determined by RT-qPCR. MTS assay was used to evaluate cell growth. The migration and invasion capacities of cells were assessed by wound healing assay and Transwell assay.

**Results:** LINC01296 was overexpressed in the TCGA-OSCC datasets. High LINC01296expression was strongly correlated with poor outcomes of OSCC patients. LINC01296 was overexpressed in OSCC tissues compared with para-carcinoma tissues. Moreover, the expression of linc01296 was higher in CAL-27, HSC-2, and SCC-25 cells than in normal human oral keratinocytes (NHOKs). Functional analysis suggested that LINC01296might be involved in the regulation of the Wnt and MAPK signaling pathways. Additionally, LINC01296 deficiency suppressed the growth, migration, and invasion of OSCC cells, whereas overexpression of TFAP2A-AS1 cause opposite results.

**Conclusion:** Our study showed that LINC01296 promoted OSCC cell growth, migration, and invasion, suggesting that LINC01296 might be a potential therapeutic target for OSCC.

## Introduction

Oral squamous cell carcinoma (OSCC) is a common malignancy with approximately 200,000 deaths and 300,000 new cases per year. It accounts for 2% of all new cancer cases and 1.9% of all cancer-related mortality worldwide. Despite great advances in OSCC treatments, the overall 5‐year survival is still below 60% 1 and the recurrence rate ranges between 18 and 76% 2, leading to a particularly poor prognosis. In addition, the early diagnosis of OSCC is difficult and the tumor is usually diagnosed at an advanced stage. The development of OSCC is a complex process involving multiple genetic changes, including aberrant expression of non-coding RNAs. The analysis of altered expression and molecular functions of non-coding RNAs may contribute to a better understanding of the pathogenic mechanisms of OSCC, as well as the development of novel cancer prevention strategies. Thus, it is important to identify new biomarkers for OSCC diagnosis or treatment.

Long non-coding RNAs (lncRNAs) are transcripts over 200 nucleotides that exhibit no or limited protein-coding potential. They are implicated in RNA stabilization, transcription regulation, remodeling chromatin, genome architecture, etc. LncRNAs play important roles in cancer development either as tumor suppressors or accelerators. The mutation and abnormal expression of lncRNA promote tumorigenesis and metastasis in many cancers 3. The dysregulation of lncRNAs also affects cancer cell invasion, metastasis, and epithelial to mesenchymal transition. Some dysregulated lncRNAs have been identified as early diagnostic markers and therapeutic targets in human cancers. LINC01296 is a member of the DUXA homeobox gene family that encodes DNA-binding proteins. The upregulation of LINC01296 promoted cancer cell proliferation and metastasis in urothelial carcinoma of the bladder 4. Wu *et al.* showed that LINC01296 promoted cancer cell proliferation and metastasis, and was associated with a poor prognosis in prostate cancer 5. However, the role of LINC01296 in OSCC remains unrevealed.

We hypothesized that LINC01296 might be involved in OSCC progression. In this study, we investigated the effects of LINC01296in OSCC using bioinformatics analysis and *in vitro* experiments.

## Materials and methods

### OSCC datasets

The RNA profiles and clinicopathological information of 382 OSCC patients and 32 healthy subjects were downloaded from the UCSC database (http://xena.ucsc.edu/). The RNA profiles of OSCC samples were normalized and summarized using the R packages “affy” and “limma”. The “limma” package was then used for differential expression analysis. The fold-change was calculated using the false discovery rate (FDR). The original *P* values were adjusted by the Benjamini-Hochberg method.

### WGCNA

WGCNA was used to analyze gene expression patterns of multiple samples using the R package “WGCNA” (https://cran.r-project.org/web/packages/WGCNA/). It can form gene modules based on similar gene expression patterns, identify the modules with highly coordinated changes, and analyze the relationship between modules and OSCC. The availability of the genes was evaluated by the “WGCNA” package. Then, an adjacency matrix was constructed to identify the correlation strength between the nodes. Subsequently, the matrix was transformed into a topological overlap matrix (TOM), which can quantitatively describe the similarity of the nodes. Hierarchical clustering was used to identify modules (minModuleSize = 30). A threshold of 0.25 was used to merge similar modules. Finally, the correlations of modules with clinicopathological information were analyzed to identify significant clinical modules.

### GSEA

GSEA is an analytical method for the interpretation of gene expression data. It focuses on genes that share common biological functions. In this study, GSEA was used to investigate potential functions and molecular mechanisms of LINC01296. OSCC patients were divided into low and high expression groups based on the median value of LINC01296 expression level, and then input into the GSEA software (http://www.gsea-msigdb.org/gsea/index.jsp).

### Functional enrichment analysis

GO and KEGG analyses are computational methods that analyze gene functions and biological pathways. The online database Metascape (http://metascape.org/gp/index.html) provides a comprehensive gene list annotation and analysis resource. GO and KEGG analyses of LINC01296-related modules were performed using the Metascape, which may improve biological understanding of these genes.

### Human tissue samples and cell lines

Seventy pairs of OSCC tissue samples and adjacent normal tissues were obtained from the Fourth Hospital of Hebei Medical University (Shijiazhuang, China). This study was approved by the ethics committee of our hospital. Written informed consent was obtained from each patient.

The OSCC cell line HSC-2, CAL-27, and SCC-25 6, 7 and normal human oral keratinocytes (NHOKs) was obtained from the Pathology Laboratory of the Cancer Institute of the Fourth Hospital of Hebei Medical University. Cells were cultured in DMEM-H medium supplemented with 10% fetal bovine serum (Thermo Fisher Scientific, Waltham, USA) and maintained in a 5% CO_2_/37 °C incubator.

### RT-qPCR

The expression of LINC01296 in tissue samples was measured by RT-qPCR. TRIzol® reagent (Solarbio Biotechnology, Beijing, China) was used to extract total RNA. GAPDH was used as an internal control. Reverse Transcription Kit (Promega, Beijing, China) was used for reverse transcription reaction. The relative expression of genes was calculated using the 2ΔΔCq method. The primers used in this experiment were as follows:

- LINC01296 forward, 5'-CGGAGAAGCAGTGGTGGGTT-3', LINC01296 reverse, 5'-GGCAGGAGAATGGCGTGAAC-3';
- GAPDH forward, 5'-TGTGTCCGTCGTGGATCTGA-3' GAPDH reverse, 5'-CCTGCTTCACCACCTTCTTGA-3'.

### Cell transfection

Small interfering RNA targeting LINC01296 (si-LINC01296, 5'-CUGAAACAUAUUCCGUGGUTT-3') and the negative control (si-control, 5'-AATTCTCCGAACGTGTCACGT-3') were constructed by GenePharma (Shanghai, China). HSC-2 cells were transfected with si-LINC01296or si-control using Lipofectamine2000 transfection reagent (Invitrogen). Transfection efficiency was evaluated after 24 h. The overexpression (OE) vector and vector control was constructed by the Gemma Gene Corporation (Shanghai, China). FuGENE® HD Transfection Reagent (Promega, Beijing, China) was used to perform the vector transfection.

### MTS assay

MTS assay was performed to assess cell viability. Cells were divided into HSC-2, si-LINC01296, and si-control groups. Cells were seeded in 96-well plates (2 × l0^4^ cells/well) and cultured for 24, 48, 72, and 96 h. Then, 20 μL of the reagent was added to cells. After 2-h incubation at 37 °C, the absorbance was detected at 492 nm using a spectrophotometer (Thermo Fisher Scientific).

### Wound healing assay

Cells were divided into HSC-2, si-LINC01296, and si-control groups. Cells transfected with lncRNA inhibitor were collected at 24 h after transfection and plated in six-well plates (5 × 10^5^ cells/well). Then, the monolayer of cells was wounded by manually scratching with a 200 µL pipette tip. The images were captured at 0, 12, and 24 h after the wound was created. The migration rate was quantified.

### Transwell migration assay

Cells were divided into HSC-2, si-LINC01296, and si-control groups. Cells transfected with lncRNA inhibitor were collected at 24-h post-transfection. For each assay, cells were plated in the upper chamber of each insert filled with serum-free medium, while 600 µL of culture medium containing 20% fetal bovine serum was added to the lower chamber. After 24 h incubation in a 5% CO_2_/37 °C incubator, cells were fixed with 4% paraformaldehyde for half an hour, stained with 0.1% crystal violet for 20 min, and then counted.

### Statistical analysis

The overall survival of OSCC patients with high or low LINC01296 expression was analyzed by Kaplan-Meier method. Log-rank test was performed to detect the difference between groups. Receiver operating characteristic (ROC) curve analysis was used to evaluate the performance of LINC01296. The correlations of LINC01296 expression with clinical characteristics were evaluated by Chi-square test. Each cell culture experiment was performed in triplicate. Data were presented as mean ± SD. The difference between two groups was analyzed by Student's *t*-test. *P* < 0.05 was considered statistically significant. Statistical analysis was performed using the R software (version 3.5.3) and SPSS 20.0 (SPSS Inc., Chicago, USA).

## Results

### LINC01296 is upregulated in OSCC

**Figure 1:**
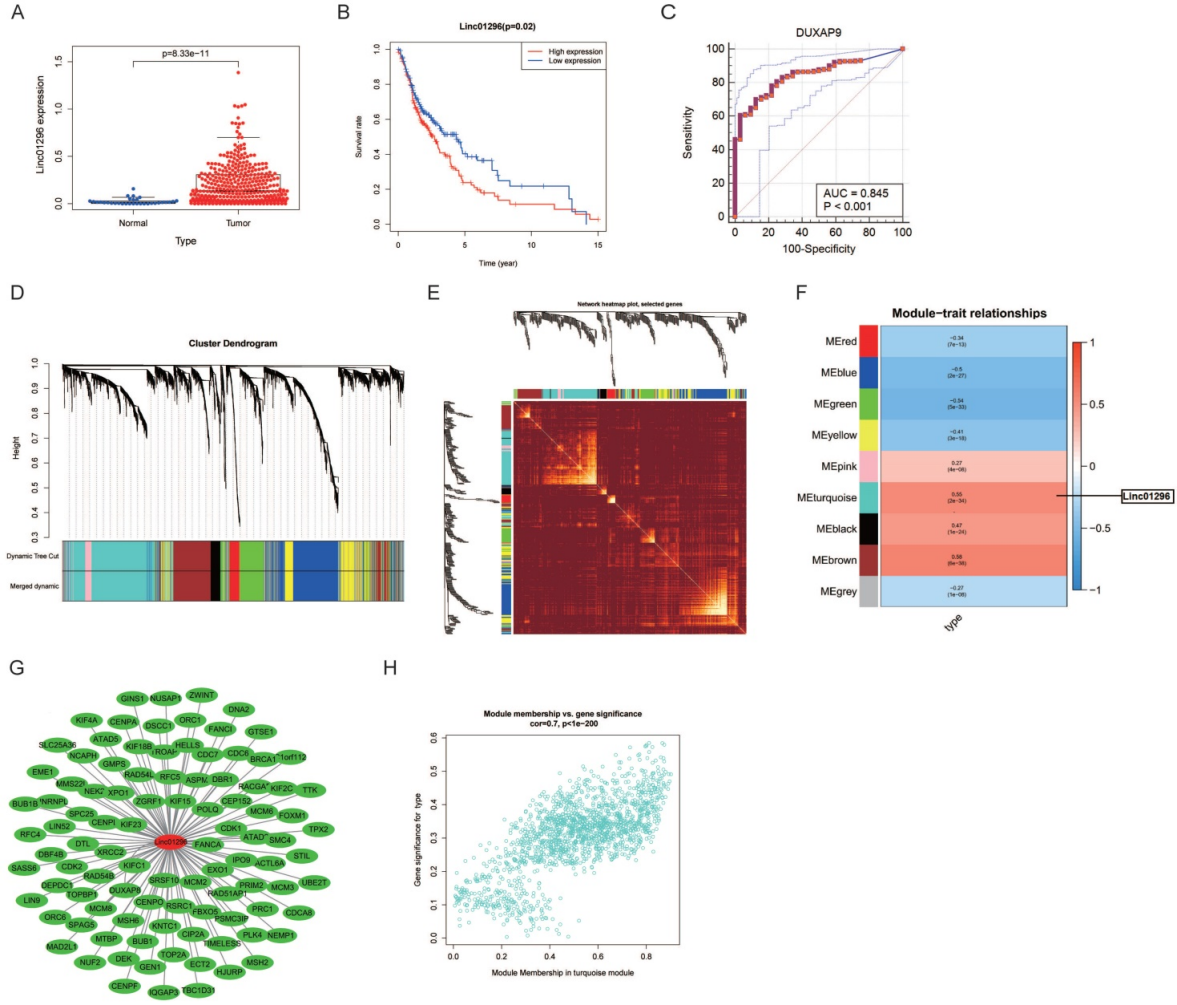
** Differential expression analysis and WGCNA analysis of the genes in the UCSC database. (A)** Box diagram of LINC01296showing differential expression between OSCC and normal groups. **(B)** Survival analysis for LINC01296in the UCSC database (P < 0.05). **(C)** ROC curve of LINC01296. **(D)** Repeated hierarchical clustering tree of all genes and LINC01296is in the turquoise module **(E)** The dendrogram and heatmap of all genes. **(F)** The associations between clinic traits and the modules and the correlation between turquoise module and OSCC is 0.55. **(G)** Interrelationships between LINC01296and the genes in the turquoise module. **(H)** Module membership in turquoise module.

The RNA profiles and clinicopathological information of 382 OSCC patients and 32 healthy subjects were download from UCSC and reanalyzed. The differential expression analysis showed that LINC01296was overexpressed in OSCC patients (*P* = 8.33e-11, Fig. 1A). The Kaplan-Meier curves showed that OSCC patients with higher LINC01296expression had worse overall survival, indicating that LINC01296might be a prognostic factor for OSCC (Fig. 1B). As shown in Fig. 1C, the area under the ROC curve was 0.845, suggesting that LINC01296might be an early diagnostic marker for OSCC patients.

The correlation between LINC01296expression and OSCC was identified by WGCNA. A hierarchical clustering tree of all lncRNAs was constructed and 9 important modules were generated (Fig. 1D). The dendrogram and heatmap of lncRNAs showed no significant differences in the interactions among modules, suggesting a high degree of independence (Fig. 1D, E). LINC01296 was in the turquoise module, and the correlation between the modules and OSCC was 0.55 (Fig. 1F), indicating that LINC01296and OSCC were related. The genes in the turquoise module are shown in Fig. 1G. The scatterplot of Gene Significance *vs.* Module Membership in the turquoise module is shown in Fig. 1H.

### GSEA and functional enrichment analysis

**Figure 2:**
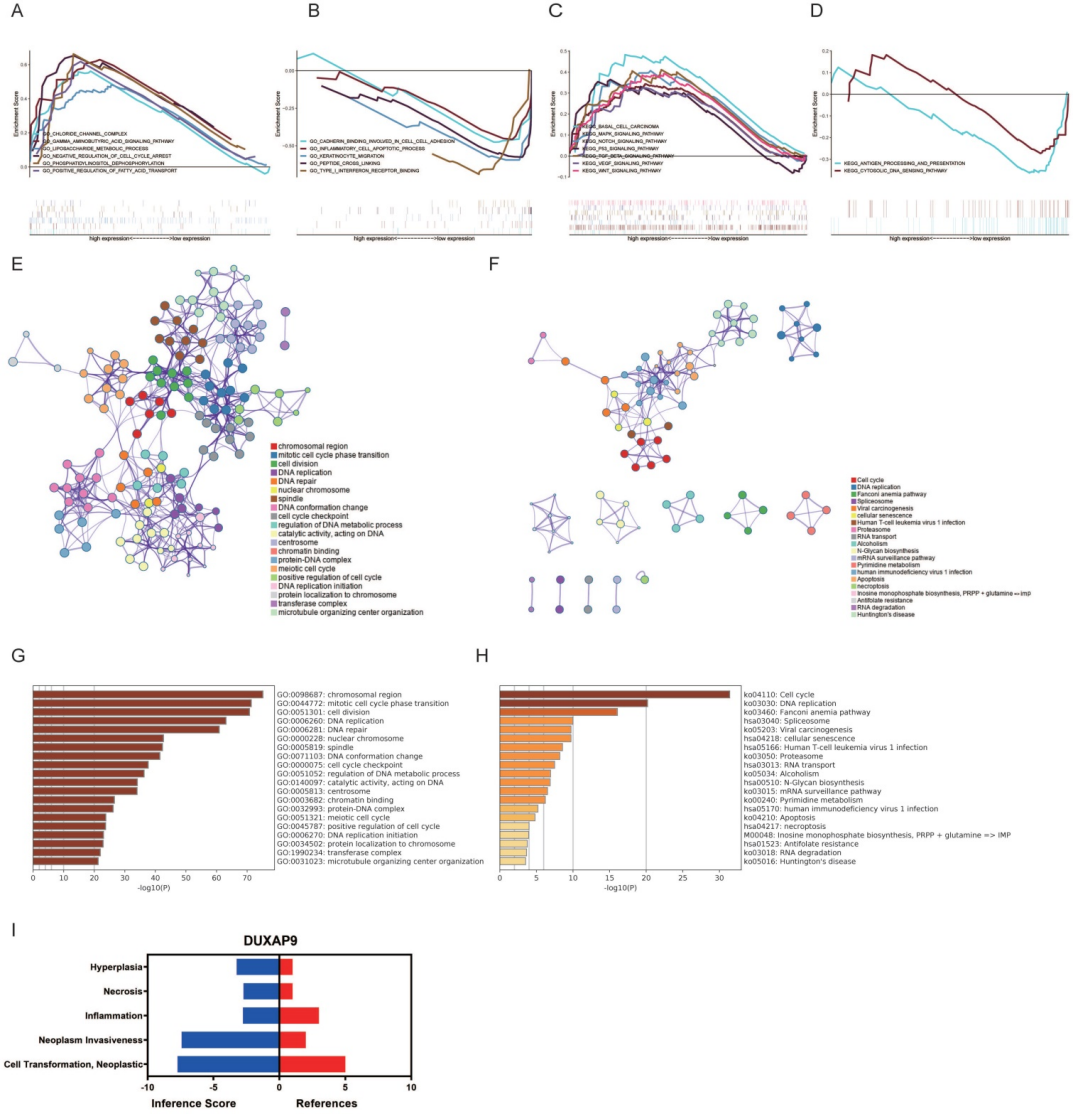
** Gene functional enrichment analysis of LINC01296. (A-B)** GO analyses by GSEA. **(C-D)** KEGG analyses of by GSEA. **(E)** GO enrichment analysis of the turquoise model genes by Metascape. **(F)** KEGG analysis **(G)** Heatmap of GO analyses by Metascape. **(H)** Heatmap of KEGG analyses by Metascape.

Next, the potential functions and molecular mechanisms of LINC01296were explored using GSEA. OSCC patients were divided into low and high LINC01296expression groups and then compared by GSEA. GO analysis showed that gamma aminobutyric acid signaling pathway, chloride channel complex, and liposaccharide metabolic process were upregulated in OSCC (Fig. 2A), while cadherin binding involved in cell-cell adhesion, inflammatory cell apoptotic process, and keratinocyte migration were downregulated (Fig. 2B). KEGG analysis revealed that the Wnt, VEGF, and MAPK signaling pathways were upregulated (Fig. 2C), whereas the cytosolic DNA sensing pathway and antigen processing and presentation were downregulated in OSCC (Fig. 2D).

GO and KEGG analyses of genes in the turquoise module were also performed. As shown in Fig. 2E, genes in the turquoise module were mainly enriched in chromosomal region, cell division, and mitotic cell cycle phase transition. The* P* values of the GO terms are shown in Fig. 2G. KEGG analysis showed that genes in the turquoise module were mainly enriched in DNA replication, the Fanconi anemia pathway, and cell cycle (Fig. 2F). The *P* values of the KEGG terms are shown in Fig. 2H.

### The expression of LINC01296 in clinical OSCC samples

**Figure 3:**
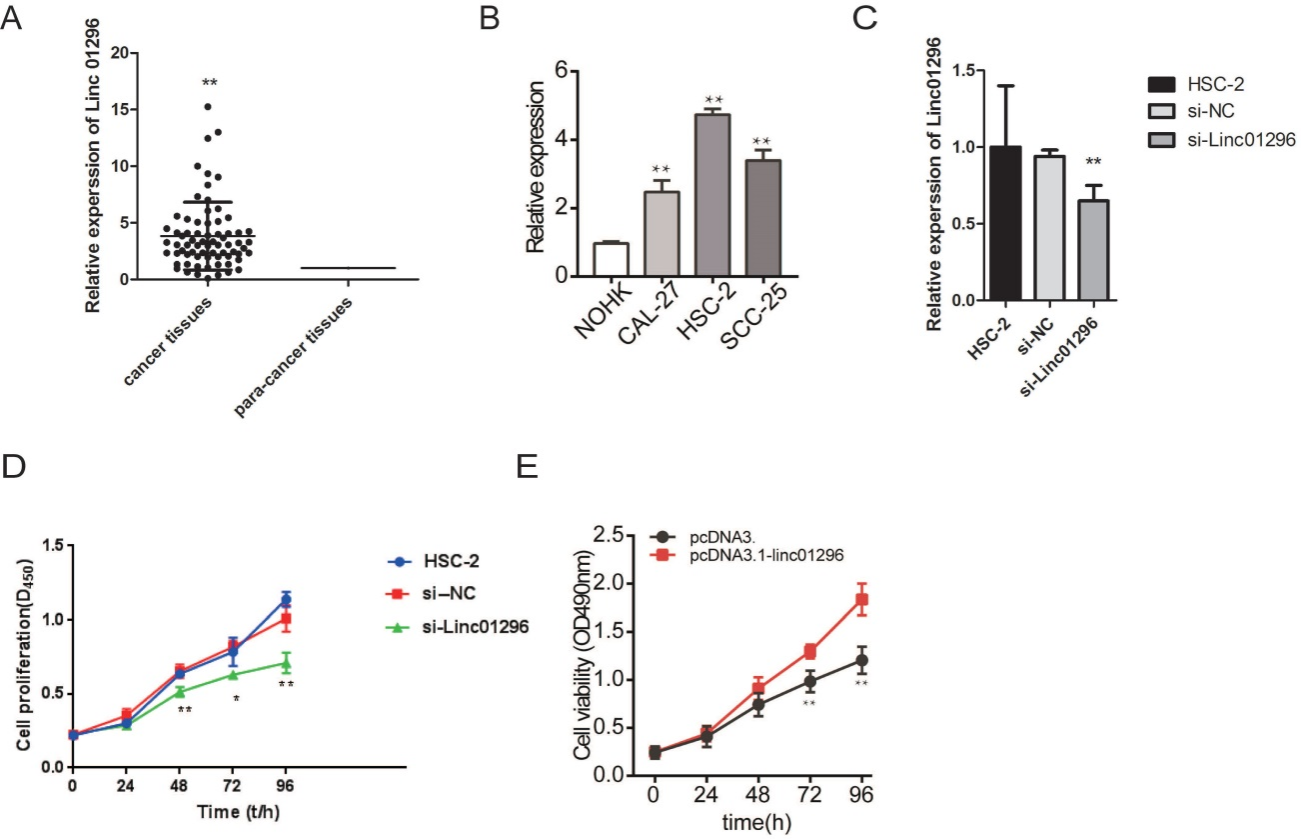
** MTS analysis is used to study the effect of inhibition of LINC01296 on cell proliferation in the HSC-2 cell line. (A)** The expression levels of LINC01296in the Clinical OSCC samples which included 70 OSCC tissues and 70 paired tissues from the adjacent normal tissues. The expression level in the adjacent tissues is set as a reference value of 1. **(B)** The expression of LINC01296is detected by qRT-PCR in HSC-2 cells. **(C)** Cell proliferation was measured by MTS assay in HSC-2 cells. Data are expressed as the mean ± SD from three independent experiments. * *P* < 0.05, ** *P* < 0.01.

We further measured the expression of LINC01296in 70 pairs of OSCC tissue samples and matched adjacent normal tissues. The overexpression of LINC01296 was frequently observed in OSCC (Fig. 3A). Moreover, LINC01296 was overexpression in OSCC cell lines than NHOKs (Fig. 3B). The correlations of LINC01296expression with clinicopathological characteristics (i.e. age, gender, pathological stage, and lymphatic metastasis) are summerised in Table 1. LINC01296expression was associated with lymphatic metastasis and pathological stage in OSCC (both *P* < 0.01, Table 1).

### LINC01296 promoted HSC-2 cell proliferation, migration, and invasion

**Figure 4:**
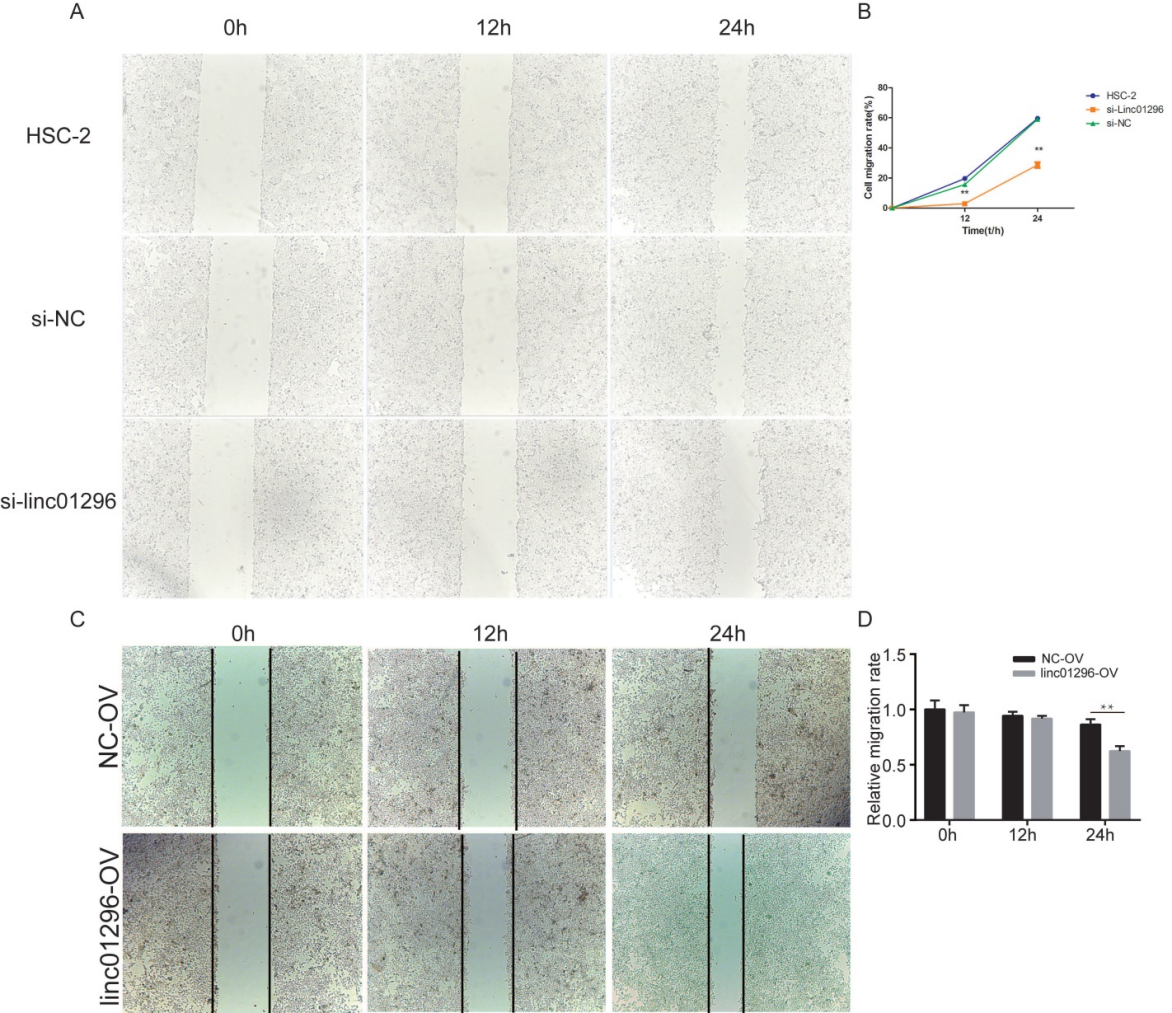
** Inhibiting the expression of LINC01296can more effectively inhibit migration of HSC-2 cells. (A)** Wound healing assay. **(B)** Wound healing assay demonstrated that silencing of LINC01296 reduced the percentage of wound closure.

**Figure 5:**
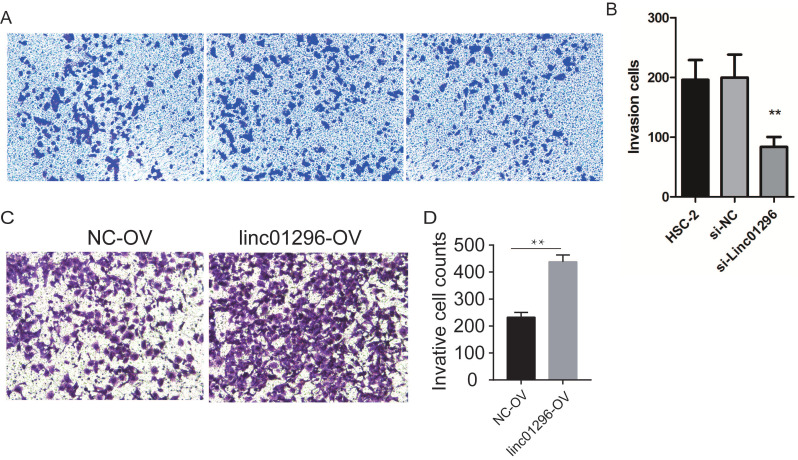
** Inhibiting the expression of LINC01296can inhibited cell invasion in HSC-2 cells. (A)** HSC-2 group, **(B)** si-control group, **(C)** si-LINC01296 group, **(D)** the transwell assay suggested that silencing of LINC01296inhibited cell invasion in HSC-2 cells.

To investigate the functions of LINC01296in OSCC, we transfected HSC-2 cells with si-LINC01296 or the negative control. The transfection with si-LINC01296 significantly downregulated LINC01296 in HSC-2 cells (Fig. 3C). The growth of HSC-2 cells was also noticeably inhibited after the transfection with si-LINC01296 (Fig. 3D), while LINC01296 overexpression could promote cell growth (Fig. 3E). Wound healing assay and Transwell assay were performed to evaluate the effects of LINC01296on cell migration and invasion. The silencing of LINC01296 significantly reduced the percentage of wound closure, whereas overexpression of LINC01296 increased the percentage of wound closure (Fig. 4). LINC01296 silencing markedly reduced the amount of crystal violet-stained cells in Transwell assay, and overexpression of LINC01296 increased the amount of crystal violet-stained cells (Fig. 5A-D). These data suggested that the silencing of LINC01296 inhibited OSCC cell invasion and migration. Taken together, LINC01296 promoted HSC-2 cell proliferation, migration, and invasion, therefore might be a therapeutic target for OSCC.

## Discussion

Oral cancer is a malignancy occurring in the oral cavity and has been considered a serious health problem resulting in high mortality and morbidity. OSCC accounts for over 90% of all oral cancers. Multimodality therapies, including surgery, radiotherapy, chemotherapy, and biologic therapy, are currently available for OSCC patients 8, 9. Although many efforts have been made to treat OSCC, the survival rate remains low over the past few years. Delayed diagnosis is one of the major reasons for low survival in OSCC 10. Thus, it is necessary to identify novel targets for early diagnosis and optimal therapies for OSCC.

The dysregulation of lncRNAs is implicated in many diseases, including cancer. Fang *et al.* found that aberrantly expressed lncRNAs were involved in chromatin remodeling, transcriptional and post-transcriptional regulation in human malignancies 11. The importance of lncRNAs in cancer development may be related to their abilities to regulate cell functions, such as cell proliferation, migration, and apoptosis 12, 13. Many lncRNAs are aberrantly expressed and act as either oncogenes or tumor suppressors in OSCC 14, 15. These lncRNAs may be used as disease-specific biomarkers and therapeutic targets for OSCC. LINC01296 belongs to the DUXA homeobox gene family. It encodes DNA-binding proteins and plays a key role in several types of cancers. Hu *et al.* reported that LINC01296 acted as an oncogene in the tumorigenesis and development of non-small cell lung cancer (NSCLC) 16. LINC01296 is also involved in the progression of colorectal cancer via regulating O-glycosylated MUC1 17. Hence, we hypothesized that LINC01296 might play a regulatory role in OSCC.

Here, we investigated the functions of LINC01296 in OSCC using data mining, bioinformatics analysis, and cell culture experiments. The results showed that LINC01296was overexpressed in the TCGA-OSCC datasets. High LINC01296 expression was correlated with poor prognosis in OSCC. Furthermore, LINC01296 was upregulated in OSCC tissue samples and its expression was associated with pathological stage and lymphatic metastasis. The knockdown of LINC01296 in HSC-2 cells significantly suppressed cell proliferation, migration, and invasion. Hence, LINC01296 might be an early diagnostic marker and a therapeutic target for OSCC.

In this study, genes in the turquoise module were obtained. They showed similar gene expression patterns as LINC01296. Cyclin dependent kinase 1 (*CDK1*) encodes a protein that belongs to the Ser/Thr protein kinase family. It is essential for G2/M and G1/S phase transitions in eukaryotic cells. CDK1 is overexpressed in lung adenocarcinoma and squamous cell lung carcinoma, and plays a key role in cancer progression 18. Liang *et al.* found that the downregulation of LINC01296repressed cell cycle progression of hepatocellular carcinoma (HCC) cells via regulating CDK1 19. Further investigations are needed to explore whether LINC01296 exerts an oncogenic role in OSCC through the *CDK1* gene. Minichromosome maintenance complex component 2 (*MCM2*) encodes a highly conserved minichromosome maintenance protein that is essential for the initiation of eukaryotic genome replication. The expression of MCM2 in HCC tissues was markedly higher than that in non-tumorous liver tissues. MCM2 plays a key role in HCC cell proliferation 20. Moreover, Yang *et al.* showed that MCM2 was intimately related to NSCLC cell proliferation and motility 21. Our data showed that MCM2 was in the turquoise module and had similar gene expression patterns as LINC01296. Thus, LINC01296 might regulate OSCC cell proliferation by targeting MCM2. This hypothesis needs to be examined in future studies with a large sample size. Replication factor C subunit 5 (*RFC5*) encodes the smallest subunit of the replication factor C complex, a protein complex required for DNA replication. RFC5 is biologically active in multiple malignant tumors and regulates the proliferation, invasion, progression, and metastasis of cancer cells 22. RFC5 was greatly upregulated in lung cancer tissues, and high RFC5 expression was associated with aggressive malignant features 23. In this study, RFC5 was in the turquoise module and had similar gene expression patterns as LINC01296. We speculated that RFC5 might also be a prognostic biomarker for OSCC.

Cell proliferation is a key characteristic of cancer development. Our data showed that LINC01296 promoted OSCC cell proliferation. Moreover, the MAPK signaling pathway was significantly enriched in GSEA. The activation of the MAPK signaling is essential for cancer cell proliferation 24-26. Further investigations with a large sample size are needed to examine whether LINC01296enhances OSCC cell proliferation by regulating the MAPK signaling pathway. We also showed that LINC01296 promoted OSCC cell migration and invasion. It has been reported that lncRNA AC007271.3 promoted the invasion of OSCC cells through mediating the Wnt/β-catenin signaling 14. Here, we showed that the Wnt signaling pathway was significantly enriched in GSEA. Thus, we hypothesized that LINC01296 promoted OSCC cell migration and invasion via activating the Wnt signaling pathway.

In conclusion, our study demonstrated the tumor-promoting effect of LINC01296 in OSCC, suggesting that the potential use of LINC01296 as a therapeutic target and an early diagnostic marker for OSCC.

## Figures and Tables

**Table 1:** Clinicopathological variables and the expression of Linc01296

Parameters	n	Linc01296		
		Low	High	P-value
**Age/year**				0.2379
>60	46	26	20	
≤60	24	10	14	
**Gender**				0.1933
Female	26	16	10	
Male	44	20	24	
**Lymphatic metastasis**				0.0000
yes	28	2	26	
no	42	34	8	
**Stage**				0.0002
I-II	48	32	16	
III-IV	22	4	18	
